# On the Present State of Medicine in Italy

**Published:** 1837-04

**Authors:** 


### ON THE PRESENT STATE OF MEDICINE IN ITALY.

In the absence of a more formal Report, we are happy to lay before our readers the following extract of a letter, which we have just received from a very intelligent friend, a physician, who has been some time resident in Italy. We hope soon to present them with a more detailed account, both of the doctrines and practice of the Italians.

"From what I have seen of the Italian physicians, I would remark, that they display considerable learning and much acquaintance with ancient authors, yet in their practice they do not appear to advance beyond the days of Hippocrates or Galen. Following closely the footsteps of the great Father of Medicine, they carefully watch the natural progress of the complaint, but unfortunately they do little either to assist or counteract the operations of nature. In their practice they seldom employ anything like decision or vigorous measures to cut short disease; and, even in the most acute complaints, they depend more on diet and regimen than on the use of medicines. I would not assert that they lose more patients than the English physicians; but I have no hesitation in saying that the bad effects of their mode of treatment is apparent in the immense number of chronic diseases which are continually presenting themselves, and which might probably have been prevented from becoming such, had depletion been more freely resorted to in the acute stage of the disease. Chronic gastritis, for example, with all the melancholy symptoms of dyspepsia which so frequently accompany this disease, and chronic enlargement and induration of the abdominal viscera, are everywhere to be seen.

"There is probably no point on which the continental practitioners differ from the English more than in the use of the lancet. Where an English physician would take away some pounds of blood to cut short an acute inflammation, an Italian takes away six or eight ounces, and repeats this venesection for days, until the disease terminates. Their ideas on this subject are very different from ours: we think that, the sooner we cut short an inflammation, the greater is our chance of preventing disorganization; they, on the contrary, believe that, by merely assisting the efforts of nature by small bleedings frequently repeated, they will at last subdue the disease more effectually, and without injuring the patient's constitution. Some, indeed, carry this difference of opinion so far as to assert that the great number of phthisical complaints in England is owing to the large bleedings which we prescribe, by which the powers of life are lowered to such a degree as to prevent the patient from again rallying. In their treatment of fevers, they employ purgatives much less frequently than we do: gentle diaphoretics, such as James's powder in one or two grain doses, alone or in combination with small quantities of spiritus mindereri and syrup of violets, are the only medicines they have any reliance on. The tepid bath is in great use among them in all febrile complaints, unless there be an eruption on the skin or an affection of the chest. The only purgatives which are used in fevers are magnesia and castor-oil, the former most frequently; but, as I said before, they do not consider it so necessary as we do to clear out the primæ viæ. When they apply blisters in inflammatory complaints,

(e. g. in pulmonic affections,) it is seldom over the part affected, but generally to the arms or legs.

"When they give medicines, they seldom, as in England, write out a prescription composed of a variety of ingredients, with directions for use: they generally write the name of one medicine on a piece of paper in the vernacular language, and give verbal directions as to the way in which it is to be employed. Indeed, their manner of prescribing is altogether so loose and irregular that one cannot help feeling that they do not place much reliance on what they prescribe.

"In regulating the patient's diet, however, they act with great judgment: here, indeed, they exhibit much more discrimination than the generality of English practitioners; and we may safely say that they are more indebted to their judicious management of the diet than to any medicine they give, for the cures they effect. In fevers and inflammations, not only everything like solid food is withheld from the patient, but even the weakest broths are forbidden; lemonades, or asses' milk, diluted with two or three parts of water, is all they allow, sometimes for twenty days, in fever; and it is only when the strength is giving way, or where the disease has nearly disappeared, that weak chicken broth would be permitted. In their theory of disease, they evidently lean more to the humoral pathology than to any of the more modern doctrines. This is remarkably the case in their treatment of chronic complaints, nervous affections, &c.; a great proportion of which they attribute to diseased humours in the blood, arising from repelled eruptions; and, for purifying the blood and removing these humours, they have a variety of medicines, chiefly vegetable, which they prescribe in the spring. Decoctions of fumaria, of graminia (the Fiorin grass), and of sarsaparilla, are often given for weeks or months, with this intention. The flesh of the viper is also a favorite remedy with them in eruptive diseases.

"In describing the opinions and practice of the Neapolitan and other Italian physicians, I must observe, that it is the older physicians whom I have chiefly in view; for there are many of the juniors in the profession who are fast approaching to the French or English mode of practice."

